# Reducing Unnecessary Biopsies Using Digital Breast Tomosynthesis and Ultrasound in Dense and Nondense Breasts

**DOI:** 10.3390/curroncol29080435

**Published:** 2022-08-04

**Authors:** Ibrahim Hadadi, Jillian Clarke, William Rae, Mark McEntee, Wendy Vincent, Ernest Ekpo

**Affiliations:** 1 Medical Image Optimisation and Perception Group, Discipline of Medical Imaging Science, Faculty of Medicine and Health, The University of Sydney, Camperdown, NSW 2006, Australia; 2 Department of Radiological Sciences, Faculty of Applied Medical Sciences, King Khalid University, Abha 62529, Saudi Arabia; 3 Medical Imaging Department, Prince of Wales Hospital, Sydney, NSW 2031, Australia; 4 Discipline of Diagnostic Radiography, UGF 12 ASSERT, Brookfield Health Sciences, University College Cork, College Road, T12 AK54 Cork, Ireland; 5 Royal Prince Alfred Hospital, Sydney Local Health District, Camperdown, NSW 2050, Australia; 6 Orange Radiology, Laboratories and Research Centre, Calabar 540281, Nigeria

**Keywords:** DBT, ultrasound, benign biopsy, breast density, breast cancer

## Abstract

Aim: To compare digital breast tomosynthesis (DBT) and ultrasound in women recalled for assessment after a positive screening mammogram and assess the potential for each of these tools to reduce unnecessary biopsies. Methods: This data linkage study included 538 women recalled for assessment from January 2017 to December 2019. The association between the recalled mammographic abnormalities and breast density was analysed using the chi-square independence test. Relative risks and the number of recalled cases requiring DBT and ultrasound assessment to prevent one unnecessary biopsy were compared using the McNemar test. Results: Breast density significantly influenced recall decisions (*p* < 0.001). Ultrasound showed greater potential to decrease unnecessary biopsies than DBT: in entirely fatty (21% vs. 5%; *p* = 0.04); scattered fibroglandular (23% vs. 10%; *p* = 0.003); heterogeneously dense (34% vs. 7%; *p* < 0.001) and extremely dense (39% vs. 9%; *p* < 0.001) breasts. The number of benign cases needing assessment to prevent one unnecessary biopsy was significantly lower with ultrasound than DBT in heterogeneously dense (1.8 vs. 7; *p* < 0.001) and extremely dense (1.9 vs. 5.1; *p* = 0.03) breasts. Conclusion: Women with dense breasts are more likely to be recalled for assessment and have a false-positive biopsy. Women with dense breasts benefit more from ultrasound assessment than from DBT.

## 1. Introduction

Early detection of breast cancer is crucial to reducing mortality from the disease [1]. Population screening using mammography is the standard approach to early detection. The principal criterion for screening recommendation is patient age, although a family history of breast cancer is also a well-established risk factor for breast cancer. In the last two decades, high mammographic breast density as a risk factor has also gained significant attention in the literature due to the associated increase in breast cancer risk and the resultant reduced sensitivity of screening mammography [2]. Technological advances and the transition from film-screen to digital mammography (DM) reduced the masking effect of breast density and enhanced cancer detection [3]. However, there are still difficulties reading DM images of women with dense breasts [4]. Mammographically dense tissue has been demonstrated to increase radiologists’ suspicion during the interpretation of screening mammograms, leading to higher recall rates for women with mammographically dense breasts [5].

Most women with dense breasts recalled for assessment have demonstrated negative or benign outcomes, with false positives ranging between 73% and 97% [6,7,8,9,10]. Unnecessary recalls increase the cost of screening, may cause psychosocial harm due to false-positive alarms, and can deter women from rescreening [11,12]. Digital breast tomosynthesis (DBT) and ultrasound can mitigate the limitations associated with DM, allowing for a more detailed evaluation of breast tissue and suspicious lesions by minimising superimposition of parenchymal densities [13,14,15]. In the United Kingdom National Health Service Breast Screening Programme, DBT assessment of women recalled at DM screening led to a 33% reduction in the benign biopsy rate [16], with lower false positives across mammographic features such as mass lesions and asymmetric densities. Some studies have also shown that using ultrasound is accurate in distinguishing between benign and malignant lesions and decreases the number of benign biopsies by 34% to 60% [17,18]. However, it is unclear how DBT and ultrasound compare when assessing women recalled and whether they reduce false-positive biopsies across different breast compositions.

Studies comparing DBT and ultrasound [19,20] have focused on incremental breast cancer detection in mammography-negative dense breasts. In one study [21], the effectiveness of ultrasound screening after the combination of DM and DBT was examined. Despite the widespread concurrent use of DBT and ultrasound in many clinical settings, no study has directly compared their diagnostic efficacy in mammography-recalled women to establish optimised assessment pathways. Importantly, no published work has compared the impact of these tools in reducing unnecessary biopsies of benign lesions, across various breast densities. We hypothesised that DBT and ultrasound result in equal numbers of unnecessary biopsies. Therefore, this study aims to compare the performance of DBT and ultrasound in women recalled for assessment after a positive screening mammogram and to compare the number of unnecessary biopsies in dense and nondense breasts.

## 2. Materials and Methods

### 2.1. Study Design and Patient Selection

This is a data linkage study of women recalled for assessment following a suspicious finding on their screening mammogram. Patients were included if they were recalled for assessment after a screening mammogram, had both DBT and ultrasound assessment, and underwent breast biopsy procedures. All screening mammograms of the recalled women were read by two radiologists who interpreted the images independently from each other. Any discrepancies between these two readers were resolved by a third radiologist.

A five-tier grading system was used to describe the findings on screening mammograms based on BreastScreen Australia’s RANZCR breast imaging lesion classification system: grade 1 (normal or no abnormality); grade 2 (benign); grade 3 (indeterminate/equivocal); grade 4 (suspicious); grade 5 (malignant). Each breast was examined using two different mammographic views: the craniocaudal (CC) and the mediolateral oblique (MLO). If more mammography spot views were required, they were obtained. Women whose images were rated 3, 4, or 5 were recalled for assessment using DBT and ultrasound to confirm or rule out breast cancer. A needle biopsy was performed on breast lesions graded 3, 4, or 5 after these imaging assessment tools were assessed. Table 1 shows the baseline characteristics of women recalled for assessment.

Breast density was reported according to the Breast Imaging Reporting and Data System (BI-RADS 5th edition): BI-RADS A: “the breasts are almost entirely fatty”; BI-RADS B: “there are scattered areas of fibroglandular density”; BI-RADS C: “the breasts are heterogeneously dense, which may obscure small masses”; BI-RADS D: “the breasts are extremely dense, which lowers the sensitivity of mammography”.

All DBT images used in this study were acquired using Selenia Dimensions (Hologic Inc.). Real time B-mode and colour doppler were performed using an ACUSON S2000 Ultrasound System (HELX Evolution with Touch Control, Siemens Medical Solutions), equipped with a 12L4 linear array transducer (12–4 MHz). Both DBT and ultrasound assessment results were evaluated according to the RANZCR breast imaging lesion classification scale.

### 2.2. Statistical Analysis

We compared the performance of DBT and ultrasound in women recalled for assessment after a positive screening mammogram to test the hypothesis that there is no difference between DBT and ultrasound in reducing unnecessary benign biopsies. The comparisons were performed according to breast density (BI-RADS A, B, C, and D). Relative risks at a 95% confidence interval were calculated to establish how DBT and ultrasound decreased the likelihood of an unnecessary biopsy following screening mammography. Using needle biopsy results as the reference standard, the number of cases requiring DBT and ultrasound assessment to prevent one unnecessary biopsy was estimated to determine the likelihood of benefit. The number needed to be assessed is inversely proportional to the risk reduction [1/(absolute risk reduction)]. The ideal screening number would be 1, in which all the women recalled for assessment with benign lesions have benefited. The association between the recalled mammographic abnormalities and breast density was analysed using the chi-square independence test (χ^2^ continuity correction). The McNemar test (χ^2^ continuity correction) was used to determine the statistical significance between DBT and ultrasound. A *p*-value ≤ 0.05 was considered statistically significant. These statistical analyses were conducted via the open-source Jamovi software (2.3.0).

## 3. Results

The study included 550 mammographic lesions from 538 women, aged 40 to 94 years (mean age: 58.9, SD: ±8.94), recalled at breast cancer screening mammography between 2017 and 2019. Among the 550 lesions recalled, 60.4% were in dense breasts, and 39.6% were in nondense breasts. Breast density was found to influence recall decisions significantly. Mammographic abnormalities were more likely to be recalled when seen in dense breasts than in non-dense breasts (*p* < 0.001). The distribution of lesion types across dense and nondense breasts is shown in Table 2.

Table 3 shows that there is no difference in true negative proportions between DBT and ultrasound in nondense breasts (32.8% vs. 22%, respectively; *p* = 0.2). Conversely, in dense breasts, ultrasound showed a significantly higher proportion of true negatives than DBT (54.8% vs. 16.1%, respectively; *p* < 0.001).

Table 4 shows the potential reduction of unnecessary biopsies for DBT and ultrasound stratified according to breast density. Differences between DBT and ultrasound in terms of preventing one unnecessary biopsy are also presented. Among all recalled mammographic abnormalities, ultrasound showed greater potential to decrease unnecessary biopsies than DBT: entirely fatty (21% vs. 5%, respectively; *p* = 0.04); scattered fibroglandular (23% vs. 10%, respectively; *p* = 0.003); heterogeneously dense (34% vs. 7%, respectively; *p* < 0.001); extremely dense (39% vs. 9%, respectively; *p* < 0.001) breasts. The number of cases needing assessment to prevent one unnecessary biopsy was significantly lower with ultrasound than with DBT in heterogeneously dense breasts (1.8 vs. 7, respectively; *p* < 0.001) and extremely dense breasts (1.9 vs. 5.1, respectively; *p* = 0.03), but there were no significant differences in entirely fatty breasts (3.2 vs. 4.3, respectively; *p* = 0.65) and scattered fibroglandular densities (2.6 vs. 4.6, respectively; *p* = 0.21).

## 4. Discussion

We found strong evidence that the density of a woman’s breast significantly influences recall decisions in a population-based screening program. Mammographic abnormalities were more likely to be recalled when seen in dense breasts than in nondense breasts. We also found that a significant number of the lesions found in women with dense breasts recalled for assessment were benign, and almost double the number of benign lesions recalled in women with nondense breasts. These findings suggest that around 1 in 3 women with dense breasts recalled for assessment had an unnecessary biopsy.

Several factors affect the interpretation of two-dimensional images and may be responsible for the high number of recalls, particularly in women with dense breasts. Summation artefacts caused by the superimposition of dense tissue on benign lesions may mimic breast cancer, which may have resulted in the high rate of unnecessary recalls [15,22]. False-positive or negative recall at screening may be due to perceptual or cognitive errors caused by factors such as poor lesion visibility and subtle or atypical cancer appearances [23]. It has been shown that breast density is more likely to cause perceptual errors such as false positives and negatives due to its ability to obscure subtle lesions or create difficulty in distinguishing lesions in distracting background breast tissue [14,24,25]. Such perceptual errors and the higher of cancer incidence in dense breasts may have contributed to the high recall of women with high breast density.

Mammographic abnormalities such as calcifications, masses with indistinct, spiculated or circumscribed margins, and asymmetries are frequent features of breast cancer [15,22,25]. Mammographic features such as calcifications and discrete masses constituted the largest proportion of benign biopsies. These two mammographic features are common findings in screening programs [26,27,28]. The high false-positive biopsies of these lesion types underscore the need for studies to establish the features of these lesions associated with malignancy to inform criteria for reducing unnecessary recall. Such studies may provide reasonable thresholds for identifying true positive lesions and reduce overtesting and unnecessary biopsies of benign lesions. Another factor that may have been responsible for the higher recall of benign lesions is lesion size. Screening quality can be judged by the detection of small cancers, defined as those with a diameter of ≤15 mm. Small-sized calcifications (≤15 mm) and calcifications that cover a larger region of breast are more likely to be malignant [29,30,31]. However, the diameters of calcifications varied widely in our data. Malignancy may be established by a complex combination of lesion features including size, morphology, and shape. Studies that combine these features to predict malignancy may better inform criteria for recall and biopsy.

A major focus of our study was to examine the potential role of DBT and ultrasound in reducing unnecessary biopsies. Previous pieces of work that compared DBT and ultrasound focused on women with mammographically negative dense breasts [19,20] and showed that ultrasound has a higher false-positive rate than DBT. Our study focuses on women with mammographically suspicious findings recalled for assessment and shows that ultrasound has significantly greater potential to decrease unnecessary biopsies than DBT in all breast compositions. We found no significant difference in true negative proportions between ultrasound and DBT in nondense breasts. In dense breasts, ultrasound showed a significantly higher proportion of true negatives than DBT. We also found that the number of cases that required assessment to prevent one unnecessary biopsy was significantly lower with ultrasound than DBT in heterogeneously dense and extremely dense breasts. These findings suggest that every benign lesion in heterogeneously and extremely dense breasts being unnecessarily recalled has approximately a 50% (1 out of 2 benign lesions) chance of receiving benefit from ultrasound.

In women with nondense breasts, we found no significant difference between DBT and ultrasound in terms of the number of cases that required assessment to prevent one unnecessary biopsy. To the best of our knowledge, the current study was the first to compare DBT and ultrasound assessments of recalled lesions across dense and nondense breasts. Previous studies [15,25] that focused on ultrasound showed that mimickers of breast cancer with benign morphologic ultrasound features could be safely managed with ultrasound follow-up to establish stability and confirm benign status. In dense breasts, ultrasound was found to be a satisfactory alternative to biopsy for solid lesions with benign morphological ultrasound features because of the high negative predictive value (99.8%) [32]; this may reduce anxiety for women recalled for assessment.

Previous studies that sought to reduce unnecessary biopsies were based on DBT. In one of these studies [16] incorporating DBT into the diagnostic workup of mammographic abnormalities would have resulted in a reduction in the number of benign biopsies conducted during screening assessment. The authors reported that DBT enhances reader accuracy and confidence in judging whether mammographic abnormalities are cancerous or not, resulting in a decrease in biopsies from 69% to 36%. However, this study did not adjust for breast density and lesion characteristics. A study from the USA [33] showed that DBT has the potential to decrease unnecessary biopsies for all breast densities, with substantial reductions for women with heterogeneously dense breasts (21.3%) and extremely dense breasts (27.5%). Our study, based on an Australian population and radiologists, showed only modest potential of DBT to reduce unnecessary biopsies for women of all breast compositions: entirely fatty (5%), scattered fibroglandular (10%), heterogeneously dense (7%), and extremely dense breasts (9%). These differences may be due to the differences in study designs and recall classification criteria. Unlike the USA study, we included women recalled for assessment following a suspicious finding on their screening mammograms that were read by two radiologists who worked independently. Additionally, the RANZCR grade 3 used by BreastScreen Australia is classified as a positive finding that combines the BI-RADS 3 and BI-RADS 4A categories in the American College of Radiologists BI-RADS Atlas. These differences may have influenced the impact of DBT during assessment for recalled women.

Although ultrasound is an effective assessment tool to differentiate between benign and malignant lesions that appear suspicious on mammography, it is limited in accurately classifying calcifications. Therefore, mammography-recalled calcifications should not be wholly ruled out based on ultrasound findings. This is supported by a previous study [17] that suggested that women should be recalled for biopsy even if suspicious calcifications are considered normal during an ultrasound. This previous work also showed a decrease in the false-positive rate in screening mammography by incorporating ultrasound into the diagnostic work-up of suspicious findings. However, further studies are needed to estimate the benefit-to-harm ratio and costs of ultrasound and DBT as assessment tools.

Our study is not without limitations. First, it is a single-centre study. Second, the sample size is relatively small, and 60.4% of recalled lesions in our study were in dense breasts, representing a large proportion of recalled mammograms. Thus, a greater understanding of work-up for dense breasts might help screening programs better manage their assessment procedures and resources.

## 5. Conclusions

The mammographic breast density increases recall rates and biopsy recommendations, and women with dense breasts recalled for assessment are more likely to have a false-positive biopsy compared to those with fatty breasts. In dense breasts, ultrasound showed greater potential to decrease unnecessary biopsies than DBT, with every benign lesion in dense breasts being unnecessarily recalled having approximately a 50% chance of benefiting from ultrasound. DBT and ultrasound perform comparatively similar in reducing unnecessary benign biopsies in fatty breasts. Therefore, tailoring assessment pathways according to breast density may reduce unnecessary biopsies and anxiety in women recalled for assessment.

## Figures and Tables

**Table 1:** Characteristics of women recalled for assessment following a suspicious mammography finding.

Characteristic	No. (%)
**Age (years)**	
40–49	69 (12.8)
50–59	222 (41.2)
60–69	163 (30.3)
≥70	84 (15.7)
**Breast Density**	
Almost entirely fatty	64 (12)
Scattered areas of fibroglandular density	152 (28.2)
Heterogeneously dense	238 (44.2)
Extremely dense	84 (15.6)
**Risk Factor of Breast Cancer (Personal/Family History)**	
Yes	98 (18.2)
No	440 (81.8)

**Table 2:** Association between recall and breast density based on lesion features.

	Breast Density			
Radiologic Feature	Nondense Breasts (Number of Benign Lesions)	Dense Breasts (Number of Benign Lesions)	Total	*p*-Value
**NSD**	26 (16)	23 (10)	49	**<0.001**
**Stellate**	51 (5)	75 (7)	126	
**Calcification**	39 (18)	128 (53)	167	
**Discrete mass**	99 (32)	82 (44)	181	
**Architectural distortion**	3 (2)	24 (10)	27	
**Total**	218 (73)	332 (124)	550	

NSD: Nonspecific density; Nondense Breasts: almost entirely fatty & scattered areas of fibroglandular density; Dense Breasts: heterogeneously dense & extremely dense; Bold values indicate statistical significance at the *p*-value ≤ 0.05 level.

**Table 3:** True negative proportions of DBT and ultrasound across dense and nondense breasts.

	NO. of True Negative (%)	Negative Outcome (Biopsy)	*p*-Value	NO. of True Negative (%)	Negative Outcome (Biopsy)	*p*-Value
	Breast Density					
Assessment Tools	Nondense Breasts			Dense Breasts		
**DBT**	16 (22)	73	0.2	20 (16.1)	124	**<0.001**
**US**	24 (32.8)	73		68 (54.8)	124	

Nondense Breasts: almost entirely fatty & scattered areas of fibroglandular density; Dense Breasts: heterogeneously dense & extremely dense; DBT: digital breast tomosynthesis; US: ultrasound; Bold values indicate statistical significance at the *p*-value ≤ 0.05 level.

**Table 4:** The potential reduction of DBT and ultrasound to decrease unnecessary biopsies following screening mammography and the number of DBT and ultrasound examinations required to be assessed to prevent one unnecessary biopsy.

	Potential Reduction of DBT and Ultrasound to Decrease Unnecessary Biopsies after DM			Estimation Number of Benign Cases that Required Assessment by DBT and Ultrasound to Prevent One Unnecessary Biopsy		
Parameter	Relative Risk (95% CI)			NO. of Cases (ARR%)		
Breast Density	DBT	US	*p*-Value	DBT	US	*p*-Value
**BI-RADS A**	0.95 (0.89 to 1)	0.79 (0.70 to 0.90)	**0.04**	4.3 (23)	3.2 (30.7)	0.65
**BI-RADS B**	0.9 (0.85 to 0.95)	0.77 (0.70 to 0.84)	**0.003**	4.6 (21.7)	2.6 (66.7)	0.21
**BI-RADS C**	0.93 (0.89 to 0.96)	0.66 (0.60 to 0.73)	**<0.001**	7 (14.1)	1.8 (55.4)	**<0.001**
**BI-RADS D**	0.91 (0.85 to 0.97)	0.61 (0.51 to 0.72)	**<0.001**	5.1 (18.8)	1.9 (50)	**0.03**

BI-RADS A: almost entirely fatty; BI-RADS B: scattered areas of fibroglandular density; BI-RADS C: heterogeneously dense; BI-RADS D: extremely dense; DM: digital mammography; DBT: digital breast tomosynthesis; US: ultrasound; ARR: absolute risk reduction. Bold values indicate statistical significance at the *p*-value ≤ 0.05 level.

## Data Availability

Data are available from the authors with the permission of BreastScreen NSW, Sydney Local Health District.
